# Overexpression of CDC42SE1 in A431 Cells Reduced Cell Proliferation by Inhibiting the Akt Pathway

**DOI:** 10.3390/cells8020117

**Published:** 2019-02-02

**Authors:** Pazhanichamy Kalailingam, Hui Bing Tan, Jiun Yit Pan, Suat Hoon Tan, Thirumaran Thanabalu

**Affiliations:** 1 School of Biological Sciences, Nanyang Technological University, 60 Nanyang Drive, Singapore 637551, Singapore; kpazhanichamy@ntu.edu.sg (P.K.); hbing194@gmail.com (H.B.T.); 2 National Skin Centre, Singapore 308205, Singapore; jypan@nsc.com.sg (J.Y.P.); shtan@nsc.com.sg (S.H.T.)

**Keywords:** CDC42, skin cancer, Akt pathway, actin cytoskeleton

## Abstract

Cell division cycle 42 (CDC42), a small Rho GTPase, plays a critical role in many cellular processes, including cell proliferation and survival. CDC42 interacts with the CRIB (Cdc42- and Rac-interactive binding) domain of CDC42SE1, a small effector protein of 9 kDa. We found that the expression of CDC42SE1 was reduced in human skin cancer samples relative to matched perilesional control. Exogenous expression of CDC42SE1 but not CDC42SE1^H38A^ (mutation within CRIB domain) in A431 cells (A431^SE1^, A431^SE1-H38A^) reduced cell proliferation. Antibody microarray analysis of A431^Ctrl^ and A431^SE1^ lysate suggested that reduced A431^SE1^ cells proliferation was due to inhibition of Akt pathway, which was confirmed by the reduced P-Akt and P-mTOR levels in A431^SE1^ cells compared to A431^Ctrl^ cells. This suggests that CDC42SE1 modulates the CDC42-mediated Akt pathway by competing with other effector proteins to bind CDC42. A431^SE1^ cells formed smaller colonies in soft agar compared to A431^Ctrl^ and A431^SE1-H38A^ cells. These findings correlate with nude mice xenograft assays, where A431^SE1^ cells formed tumors with significantly-reduced volume compared to the tumors formed by A431^Ctrl^ cells. Our results suggest that CDC42SE1 is downregulated in skin cancer to promote tumorigenesis, and thus CDC42SE1 might be an important marker of skin cancer progression.

## 1. Introduction

Rho GTPases regulate diverse cellular functions and have been implicated in cancer initiation and progression, playing critical roles in cell proliferation, cell survival, invasion, and metastasis [1]. Mutations and epigenetic changes in cells are the main causes of cancer development [2]. Although mutations in Rho GTPases are rarely found in tumors, their expression levels and activity are usually dysregulated during tumor initiation and progression [3]. Rho GTPases are activated by guanine nucleotide exchange factors (GEFs), which promote exchange of GDP (inactive state) to GTP (active state), while GDI (GDP-dissociation inhibitor) prevents it. Activated Rho GTPases interact with a variety of effector proteins to regulate cellular processes, such as actin polymerization, cytoskeletal reorganization, cell migration, cell cycle progression, and cell polarity [4]. Subsequently, Rho GTPases are inactivated by GTPase Activating Proteins (GAPs), which promote hydrolysis of GTP into GDP [5]. The activity of Rho GTPases have been found to be higher in cancer cells due to alteration in the expression of GEFs, GAPs, and GDIs [6]. Previous reports suggest that diverse Rho GTPases (CDC42, RhoA, Rac, RhoV, and RhoC) are activated in various types of cancer [6].

Rho family member CDC42 regulates proteins with diverse biochemical functions [7], such as Kinases MRCKs (Myotonic dystrophy kinase-related CDC42-binding kinases), PAKs (p21-activated kinases), MLKs (mixed lineage kinases) and scaffolding proteins (par6, WASP, N-WASP, and IQGAP) [8,9,10,11]. The abnormal activation of CDC42 may alter the various downstream signaling pathways regulated by CDC42, leading to uncontrolled cell proliferation and cancer development [12]. However, under normal physiological conditions, CDC42 and its effector proteins are required for cellular functions, such as actin polymerization, cell proliferation, cell migration, and cell polarity [13]. CDC42SE1 and CDC42SE2, also referred to as SPEC1 and SPEC2 [14] (Small effector of CDC42 protein), are small CDC42 effector proteins. CDC42SE1 consist of 79 amino acids (CDC42SE2, 84 amino acids), including conserved CRIB domain, 2 conserved cysteine at the N-terminal, and a basic amino acid preceding the CRIB domain. CDC42SE1 and CDC42SE2 regulate CDC42-induced changes to cell morphology, as well as the accumulation of F-actin at immunological synapses and phagocytosis [8,14,15]. Actin cytoskeletal remodeling and actin polymerization activity play critical roles in cell motility, cell growth, cancer development, and progression, and eventually metastasis [16]. However, the role of CDC42SE1 in cell proliferation of skin cancer remains unknown.

The skin is the largest organ, consisting of the dermis and epidermis [17]. The epidermis, the outer layer of the skin, is in direct contact with the environment, including foreign particles, chemicals, pathogens, and UV rays [18]. Long term exposure of skin cells to chemicals and UV rays causes DNA mutations, potentially leading to cancerous development, consequently skin cancer is the most prevalent cancers in human, particularly among populations with low melanin in the skin [19]. Skin cancers are commonly divided into melanoma and non-melanoma skin cancer (NMSC). NMSC is further subdivided into basal cell carcinomas (BCCs) and squamous cell carcinomas (SCCs) [20]. The American Academy of Dermatology estimated that approximately 9500 people are diagnosed with skin cancer each day and that over 3 million Americans are affected by NMSC each year [21].

The PI3K/Akt signaling pathway is highly activated in BCCs and SCCs [22]. Both cytokines and growth factors (e.g., IGF-1, IL-6, and TNF-α) are involved in activating the PI3K pathway [23]. Binding of cytokines or growth factors to their receptors leads to PI3K-mediated phosphorylation of phosphatidylinositol-3,4 (PIP2) to phosphatidylinositol-3,4,5 (PIP3), and the activation of Akt by phosphorylating Thr308 in a PDK1-dependent manner [24]. In addition, the activation of Akt also requires phosphorylation in the regulatory domain of Akt (Ser473) by mTORC2 [22]. The mTORC1 complex [25] is a key downstream effector of Akt, and activated Akt phosphorylates the heterodimeric TSC1-TSC2 complex and inhibits the GAP activity of TSC1-TSC2, releasing Rheb GTPase (Ras homologue enriched in brain), which activates mTORC1 [26]. PTEN is a tumor suppressor that converts PIP3 to PIP2 by dephosphorylation, thus downregulating downstream signals of the Akt pathway. The dysregulation of PI3K/Akt signaling was found to be responsible for abnormal cell proliferation, avoidance of apoptosis, and malignant transformation [27]. Interestingly, JNK and c-Jun are highly expressed in BCCs and SCCs correlating with their tumorigenic activity [28]. The cross-talk of these several pathways, including the RAS/Raf/PI3K/Akt signaling axis, are likely responsible for abnormal cell proliferation, tumor growth, and cell transformation in skin cancers [29].

In this study, we found that the expression of CDC42SE1 was reduced in SCC samples compared to the matched perilesional controls. Overexpressing CDC42SE1 in A431 cells (A431^SE1^) significantly reduced cell proliferation, formed smaller colonies in soft agar, and smaller tumors in nude mice compared to A431^Ctrl^ cells. Overexpression of CDC42SE1^H38A^ mutant in A431 cells did not reduce the cell proliferation, suggesting CDC42SE1 interaction with CDC42 is critical to inhibit cell proliferation. Antibody microarray analysis of cell lysate from A431^Ctrl^ and A431^SE1^ and subsequent analysis of the data using Ingenuity Pathway Analysis (IPA software) suggested that the inhibition of the Akt pathway correlate with reduced cell proliferation. The inhibition of the Akt pathway was further validated by immunoblot analysis with reduced phosphorylated Akt (p-Akt) and mTOR (P-mTOR). Thus, the binding of CDC42 to the CRIB domain of CDC42SE1 caused the inhibition of Akt pathway. These results suggest that the downregulation of CDC42SE1 promotes cell proliferation and cancer progression and may be a novel marker for skin cancer development.

## 2. Materials and Methods

### 2.1. Cell Culture, Generation of Stable Cells by Lentiviral Transduction

HEK293T and A431cells were cultured in DMEM (Sigma-Aldrich, St. Louis, MO, USA) media containing 10% FBS and 1% penicillin-streptomycin at 37 °C in a humidified incubator containing 5% CO_2_. The lentivirus particles were generated by transfecting target vector pLJM1-GFP (a gift from David Sabatini (Addgene plasmid # 19319) [30] or its derivatives (pLJM-CDC42SE1-His, pLJM-CDC42SE1^H38A^-His, pLJM-CDC42SE1-GFP, pLJM-CDC42SE1^H38A^-GFP) together with third-generation packaging constructs into HEK293T cells. The culture supernatant with virus particles were collected after 24 and 48 h post transfection, filtered, and used to infect A431 cells. Puromycin (2 µg/mL) was used for selecting stable cells with virus infection.

### 2.2. Quantitative PCR

Human SCC samples from patients (n = 5) and the perilesional control (n = 5) samples embedded in paraffin sections were obtained from the National Skin Centre (NSC), Singapore. Total RNA was isolated from SCC samples, Perilesional samples, A431, HaCaT, and HSC-5 cells. The RNA was reverse transcribed to cDNA and the cDNA was used as template in quantitative PCR using a thermal cycler (BioRad, Hercules, CA, USA) and SYBR Green/ROX qPCR Master Mix (Fermentas, Waltham, MA, USA). The sense and anti-sense primer for human CDC42SE1 were 5′-CGGATTGACCGGACCATGATT and 5′-CGGTTTCCCTTGGATCTCATCT; MRPL-27 primers 5′-CTGGTGGCTGGAATTGACCGCTA and 5′-CAAGGGGATATCCACAGAGTACCTTG were used for normalization.

### 2.3. Kinex™ Antibody Microarray Analysis

A431^SE1^ and A431^Ctrl^ cells grown in a 100mm culture dish were harvested, washed with PBS, and centrifuged at 14,000× *g* for 5 min. The cell pellet was lysed with Kinex^TM^ lysate buffer (Vancouver, BC, Canada), as per the manufacturer’s protocol. Protein lysates (50 μg) from A431^SE1^ and A431^Ctrl^ cells were labeled with fluorescent dye provided in the kit. The labelled samples were loaded separately onto the antibody microarray glass slide and incubated for 2 h at room temperature. The microarray slide was washed after the incubation to remove unbound protein and scanned with a Perkin-Elmer Scan Array Express Reader (Waltham, MA, USA).

### 2.4. MTT and Cell Proliferation Assay

A431^Ctrl^, A431^SE1^, and A431^SE1-H38A^ (7500 cells/well) were seeded in a 24-well plate and incubated at 37 °C with 5% CO_2_. After 72 h incubation, the cells were used for the MTT assay and cell counting with hemocytometer. For MTT assay, the tetrazolium salt, 3-4,5-dimethylthiazol-2,5-diphenyl tetrazolium bromide (MTT) (5 mg/mL) was added to each well and incubated for 3.5 h at 37 °C in CO_2_ incubator. MTT solvent (0.1% NP-40 with 4mM HCl) was added slowly into the well and kept for 15 min. The optical density was measured using a plate reader (Tecan, Männedorf, Switzerland) at 590 nm and at 620 nm (reference). The readings at 620 nm were subtracted from the 590 nm readings.

### 2.5. Cell Spreading Assay

A431^SE1^, A431^SE1-H38A^, and A431^Ctrl^ cells (30,000 cells/well) were seeded on a fibronectin coated 96-well plate and incubated at 37 °C in a humidified CO_2_ (5%) incubator. The cells were imaged at 0 min, 10 min, and 20 min time intervals. The surface area of the cells (30 cells/well) was calculated using Image J software [31].

### 2.6. Colony Formation Assay

A431^SE1^, A431^SE1-H38A^, and A431^Ctrl^ cells (1 × 10^3^ cells/well) were seeded in a 6-well plate and cultured with DMEM with 10% FBS for two weeks. Colonies were stained with 0.05% Crystal Violet for 30 min and washed 5 times with PBS. The number of colonies (> 0.1 mm) were counted manually from three independent experiments.

### 2.7. Soft Agar Colony Formation Assay

We coated 6-well plates with 1.0% noble agar in complete media (1.5 mL agar/well) and allowed it to solidify at room temperature for 15 min. A431^SE1^, A431^SE1-H38A^, and A431^Ctrl^ cells (25 × 10^3^/mL) were separately mixed with 0.6% noble agar and added to separate agar-coated wells and allowed to solidify for another 20 min. Complete media (500 µL) was added to each well to prevent drying, and they were incubated for 14 days. Colonies were stained with 0.05% Crystal Violet for 1 h, washed with PBS, and images of the colonies were captured using an Olympus microscope (Tokyo, Japan) with 4× objective lens. The average number of colonies was calculated manually, and the average area of colonies was quantified using Image J software

### 2.8. Immunoblotting

Cells were lysed using RIPA lysis buffer (Sigma-Aldrich, St. Louis, MO, USA) and the equivalent of 30 µg of total protein was boiled with SDS-PAGE sample buffer 5 min at 100 °C, proteins were resolved using Polyacrylamide (8%, 10% or 15%) SDS-PAGE gel, and transferred onto nitrocellulose membrane. The membrane was probed with primary antibodies CDC42SE1, Akt, P-Akt, mTOR, P-mTOR, 4EBP-1, P-4EBP-1, P-PTEN, PTEN, washed, incubated with secondary antibody conjugated to HRP, washed, and developed using Immobilon^TM^ Western blot reagent (WBKLSO500, Millipore, Burlington, MA, USA).

### 2.9. Immunofluorescence

Cells (30 × 10^3^ cells/coverslip) cultured on coverslips in a 6-well plate were fixed with 4% PFA (paraformaldehyde) for 15 min, permeabilized with 0.2% PBST (PBS with 0.2% Triton X-100) for 20 min, and blocked with 1% BSA in PBS for 30 min. Cells were incubated for 1 h with primary E-cadherin antibody (1:50) in 1% BSA in PBS. Cells were washed (5 times) with 0.1% PBST and were incubated with Alexa488-conjugated goat anti-mouse antibody for 1 h. Cells were washed 5 times with 0.1% PBST. Cells were subsequently stained with Alexa568-Phalloidin for 1 h and DAPI for 15 min. Images were acquired using Olympus microscope with Photo metrics Cool Snap HQ2 camera (Photometrics, Tucson, AZ, USA). The fluorescence intensity of E-cadherin staining of each cell was quantified using Image J software (Version 1.41, n = 30). We used freehand icon from Image J to draw an ROI (Region of Interest) encompassing the plasma membrane of cells and quantified the fluorescence intensity, and divided by 2 if the ROI covered plasma membranes from two cells.

### 2.10. Filopodia Analysis

A549 cells were seeded (60,000 cells/well) in a 6-well plate with a 18 mm coverslip and cultured overnight. Cells were than transfected with either of the following plasmid mixtures (1 µg + 1.5 µg + 1.5 µg) according to our grouping as follows: (1) 1 µg pGFP + 1.5 µg pCDC42^G12V^ + 1.5 µg Vect, (2) 1 µg pGFP + 1.5 µg pCDC42^G12V^ + 1.5 µg pCDC42SE1, (3) 1 µg pGFP + 1.5 µg pCDC42^G12V^ + 1.5 µg pCDC42SE1^H38A^. The coverslip was removed 36 h post-transfection and placed on a microscope slide to visualize filopodia using a 40× objective fitted Olympus microscope with Photo metrics Cool Snap HQ2 camera. For each experiment, we used 30 transfected cells to quantify the number of filopodia per cell (membrane projections between 8 and 20 μm).

### 2.11. Xenograft

Stable cells A431^SE1^ and A431^Ctrl^ cells (1 × 10^6^ cells/50 μL) in cold DMEM were mixed with 50 μL of Matrigel and injected subcutaneously into nude mice (approved by The NTU IACUC, ARF-SBS/NIE-A0325). Tumor dimensions (Length, L and Width, W) were measured using a Vernier caliper (Fujian, China) at the 8th, 11th, 15th 18th, and 21st day post- injection and the tumor volume was calculated using L X W^2^/2. Mice were sacrificed at the end of 22nd day post-injection.

### 2.12. Histology and Immunofluorescence Staining

Mice were anesthetized and sacrificed with CO_2_ inhalation. Tumors were removed from the skin and fixed in 4% (PFA) overnight at 4 °C. Fixed tumor samples were washed with 1× PBS and then dehydrated by sequential 1 h incubation in 70, 80, 90, and 100% ethanol. Next, samples were incubated in 50% xylene/ethanol mixture followed by incubation in pure xylene. Dehydrated samples were then submerged overnight in paraffin wax at 60 °C and subsequently embedded in paraffin molds. Paraffin embedded tissue was sectioned (5 μm) and transferred onto superfrost slides (Fisher Scientific, Bellefonte, PA, USA). The slides were kept at 60 °C for 3 h to remove the paraffin and subsequently rehydrated with pure xylene, 50% xylene/ethanol mixture, 100%, 90%, 80%, 70%, and 60% ethanol for 5 min each, and stained with hematoxylin and eosin (H & E) as described [32]. For immunostaining, tumor slides were blocked with 1% BSA for 45 min and incubated with anti-CD31 primary antibody (#ab-28364, Abcam, Boston, MA, USA) overnight at 4 °C. Slides were then incubated with secondary antibodies conjugated with Alexa fluor 488 for 1 h at room temperature. Nuclei were visualized with DAPI staining for 15 min. Then, slides were washed with 1× PBS and mounted with DPX mounting media. The images were acquired using Olympus microscope with Cool Snap HQ2 camera.

### 2.13. Statistical Analysis

Statistical analysis was performed using student *t*-test, and *p*-values ≤ 0.05 were considered statistically significant from three independent experiments. Values presented in bar charts represent mean ± SD.

## 3. Results

### 3.1. CDC42SE1 Expression Is Reduced in Skin Cancer

CDC42 is a Rho GTPase and a key regulator in cancer growth, proliferation, survival, and in metastasis [13]. CDC42 binds to CRIB domains of effector proteins to regulate the actin cytoskeleton and cell polarity in mammalian cells [33]. CDC42SE1 is a small effector of CDC42 and their function in cancer remains unknown.

**Figure 1:**
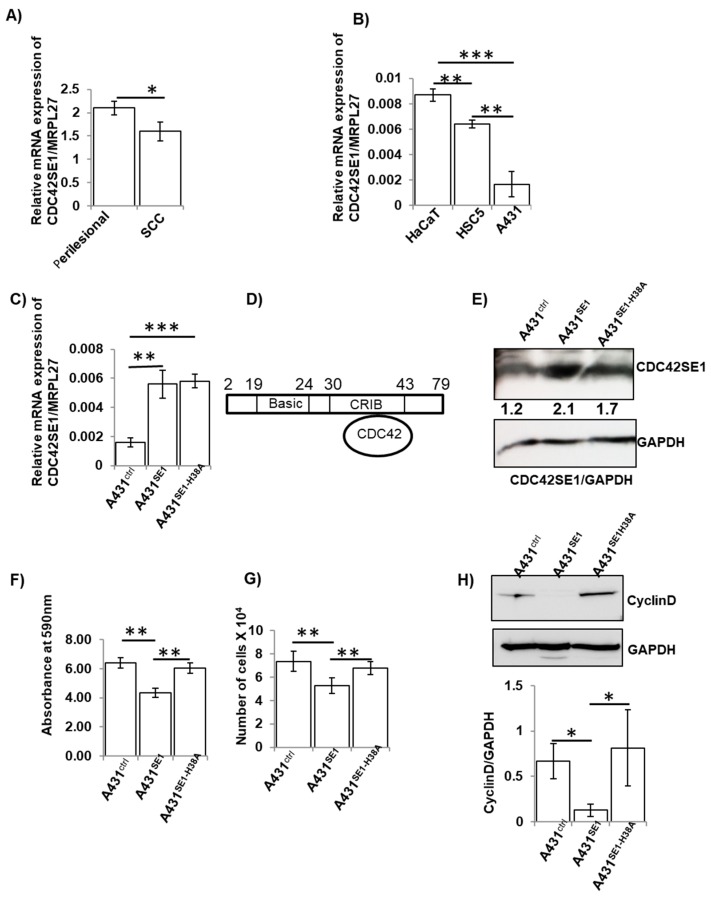
Expression of CDC42SE1 is reduced in SCC samples and exogenous expression of CDC42SE1 reduced cell proliferation. (**A**) Quantitative PCR analysis of expression of CDC42SE1 in SCC biopsies (n = 5) vs. the matched perilesional controls. Expression of MRPL27 was used to normalize. (**B**) Quantitative PCR analysis of expression of CDC42SE1 in HaCaT, HSC5, and A431 cells. Expression of MRPL27 was used to normalize. (**C**) Quantitative PCR analysis of expression of CDC42SE1 in A431^Ctrl^, A431^SE1^, and A431^SE1-H38A^ cells (n = 3). Expression of MRPL27 was used to normalize. (**D**) Structure of CDC42SE1 with CDC42 binding. (**E**) Immunoblot analysis of CDC42SE1 expression in A431^Ctrl^, A431^SE1^, and A431^SE1-H38A^ cells. GAPDH (Glyceraldehyde 3-phosphate dehydrogenase) were used as loading control (n = 3). (**F**) MTT assay and (**G**) cell proliferation assay of A431^Ctrl^, A431^SE1^, and A431^SE1-H38A^ cells (n = 3). In total, 7500 cells were seeded in a 24-well plate and incubated at 37 °C with 5% CO_2_. After 72 h incubation, cells were used for MTT assay and cell counting with hemocytometer. (**H**) Protein lysate from A431^SE1^, A431^SE1-H38A^, and A431^Ctrl^ were subjected to western blot using anti-CyclinD1 and GAPDH was used as a loading control (n = 3). Note: *** *p* ≤ 0.001, ** *p* ≤ 0.01, * *p* ≤ 0.05.

In order to characterize the role of CDC42SE1 in skin cancer, we analyzed the expression of CDC42SE1 in the SCC samples and matched perilesional controls (n = 5) using qPCR (Figure 1A). The expression of CDC42SE1 was significantly reduced in human SCC samples (n = 5) compared to matched perilesional controls (n = 5) (Figure 1A). We analyzed the overall survival and expression of CDC42SE1 in head-neck squamous cell carcinoma (n = 259) using the Kaplan-Meier Plotter (http://kmplot.com/analysis/) [34], a database which integrates clinical and gene expression data (Figure S1). We found that patients with low expression of CDC42SE1 died faster compared to patients with high expression of CDC42SE1. These results corroborated our hypothesis. To identify an in vitro model, we checked for the expression of CDC42SE1 in human immortalized keratinocytes (HaCaT) [35], HSC5 (human skin squamous cell carcinoma cell line) [36], and A431 (Epidermoid carcinoma cell line) [37] cell lines. The expression of CDC42SE1 was significantly higher in HaCaT cells compared to HSC5 and A431 cells, with A431 cells having the lowest expression among the three cell lines (Figure 1B). Thus, the results indicate that CDC42SE1 is highly expressed in normal keratinocytes while being reduced in carcinoma cell lines. These results suggest that the expression of CDC42SE1 is reduced during tumorigenesis. In order to characterize the role of CDC42SE1 in skin cancer and to identify the signaling pathways regulated by CDC42SE1, we focus our effort on the A431 cell line.

### 3.2. CDC42SE1 Inhibits Cell Proliferation and Growth of A431 Cells in Soft Agar

CDC42 effectors play a critical role in different cancers, either by activating or inhibiting signaling pathways which regulate cell proliferation [38]. A previous study proposed that CDC42SE1 inhibits the CDC42-induced JNK activity [14] and cell morphological changes in COS1 cells and NIH-3T3 fibroblasts [14]. However, the role of CDC42SE1 in skin cancer has not been characterized. The interaction of CDC42SE1 with CDC42 is mediated through the CRIB domain, and H38A mutation within the CRIB domain is sufficient to disrupt this interaction (Figure 1D) [14]. In order to characterize the role of CDC42SE1 in the proliferation of skin cancer, we generated three A431 sublines, by infecting A431 cells with lentivirus generated using empty target vector (A431^Ctrl^), vector expressing CDC42SE1 (A431^SE1^), and vector expressing CDC42SE1^H38A^ (A431^SE1-H38A^), and used them for in-vitro analysis. The successful overexpression of CDC42SE1 (A431^SE1^) and CDC42SE1^H38A^ (A431^SE1-H38A^) in A431 cells was validated by qPCR and immunoblot (Figure 1C,E). In order to determine the role of CDC42SE1 in cell proliferation, we carried out MTT assay and cell proliferation assay. The proliferation of A431^SE1^ cells was significantly reduced compared to A431^Ctrl^ and A431^SE1-H38A^ cells (Figure 1F,G).

**Figure 2:**
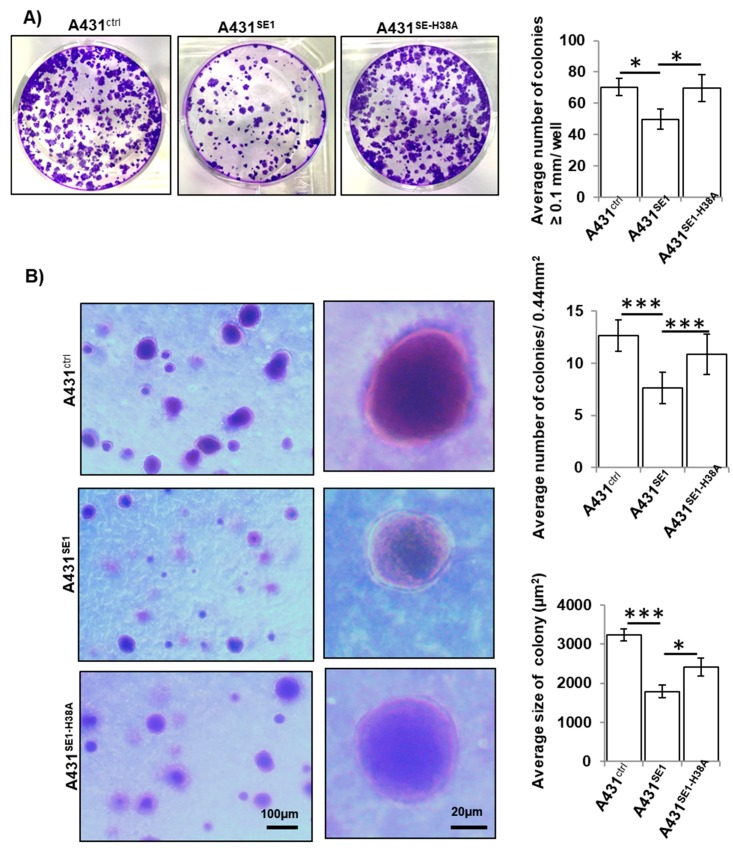
CDC42SE1 inhibits colony formation and growth of A431 cells in soft agar. (**A**) A431 cells (A431^Ctrl^, A431^SE1^, and A431^SE1-H38A^) cells were grown for 14 days before being stained with 0.05% crystal violet and imaged. Graph represents the average number of colonies/well. (**B**) A431 cells (A431^Ctrl^, A431^SE1^, and A431^SE1-H38A^) were grown in 0.6% agar for 14 days and stained with 0.05% crystal violet and imaged. Graph represents average the number of colonies/area and average size of colonies. Images were taken with a 5× objective and 6 fields were counted for each sample (n = 3). Colony area (µm^2^) was measured using ImageJ software. (*** *p* ≤ 0.001, * *p* ≤ 0.05).

Proliferation of A431^SE1-H38A^ cells was higher than A431^SE1^ cells and was comparable to that of A431^Ctrl^, suggesting that the CDC42-CDC42SE1 interaction is critical for the attenuation of cell proliferation. In addition, we observed a drastically reduced expression of cyclin D1 in A431^SE1^ cells compared to A431^Ctrl^ and A431^SE1-H38A^ cells (Figure 1H). Cyclin D1 plays a crucial role in cell cycle progression and cell proliferation. An elevated level of cyclin D1 during the G2 phase promotes cell proliferation, while the degradation and reduced cyclin D1 levels are associated with attenuated cell proliferation [39]. Together, it suggests that reduced Cyclin D1 is correlated with reduced cell proliferation in A431^SE1^ cells compared to A431^Ctrl^ and A431^SE1-H38A^ cells. Similarly, we observed a reduction in colony size and number formed by formed by A431^SE1^ cells compared to A431^Ctrl^ and A431^SE1-H38A^ cells (Figure 2A), suggesting inhibition of tumor initiation and cell survival in A431^SE1^ cells compared to A431^Ctrl^ and A431^SE1-H38A^ cells.

Anchorage-independent growth is one of the hallmarks of cellular transformation and is an accurate in-vitro assay for detecting malignant transformation of cells [40]. Thus, we examined the effect of CDC42SE1 in tumorigenic activity of A431 cells using anchorage independent growth assay. We grew the three A431 cell lines in soft agar for 14 days and quantified the number of colonies and size of the colonies formed. The overexpression of CDC42SE1 caused a significant decrease in the anchorage independent growth of A431^SE1^ cells, as we observed a decrease in colony size and number on soft agar compared to A431^SE1-H38A^ and A431^Ctrl^ cells (Figure 2B). Previously it was reported that co-transfection of CDC42SE1 with CDC42 in COS1 cells led to the inhibition of CDC42-induced JNK expression [14]. Thus, our finding that overexpression of CDC42SE1 in A431 caused reduced cell proliferation, colony formation, and anchorage independent growth of A431 cells could be due to downregulation of CDC42 mediated signaling pathway by binding of CDC42SE1 via its CRIB domain.

### 3.3. CDC42SE1 Regulates Cell Proliferation through the Akt Pathway in A431 Cells

**Figure 3:**
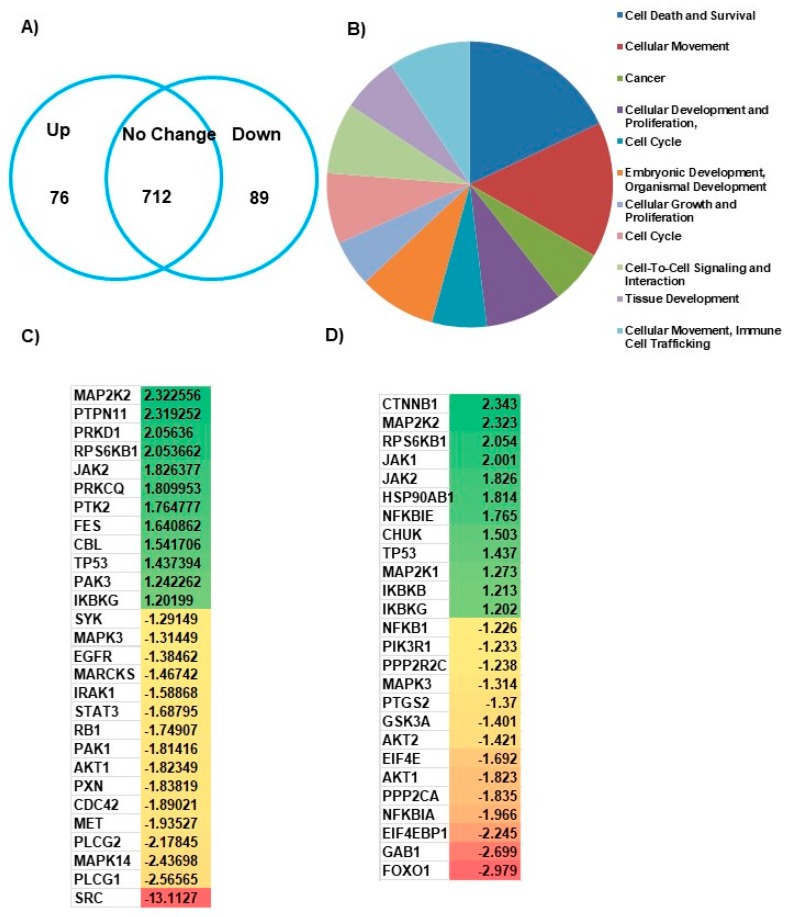
CDC42SE1 regulates cell proliferation through Akt pathway in A431 cells. (**A**) A431^SE1^ and A431^Ctrl^ cell lysates were labelled and analyzed using Kinex™ antibody array, which interrogated expression levels or phosphorylation status of 877 cell signaling proteins. Protein expression was upregulated by ≥ 1.2-fold or downregulated by ≤ 1.2 is presented in the Venn diagram. The fold changes in expression were calculated using Ingenuity Pathway Analysis (IPA). (**B**) Pie chart representing gene ontology classification of Kinex™ antibody array based on biological functions. (**C**) The heat map diagram represents the inhibition of neoplastic cell growth in data generated from A431^SE1^ and A431^Ctrl^ using IPA software. (**D**) The heat map diagram represents the Akt pathway proteins, which are differentially expressed in A431^SE1^ cells compared to A431^Ctrl^ cells, as predicted by IPA software.

To identify the molecular mechanism responsible for the CDC42SE1-mediated attenuation of A431 cells proliferation, we performed an antibody microarray analysis of protein lysate from A431^SE1^ and A431^Ctrl^ cells. The antibody microarray interrogates levels of 518 proteins and 359 phosphoproteins (Figure 3A). To identify pathways that are dysregulated by the overexpression of CDC42SE1, we performed functional annotation using the gene ontology (GO) software, IPA (Ingenuity Pathway Analysis). Gene ontology annotated differentially expressed genes according to biological process, and the top 5 ranked processes, which are dysregulated in A431^SE1^, are cell death and survival, cellular migration, cancer, cellular development and proliferation, and cell cycle (Figure 3B). The cell proliferation gene ontology showed genes involved mainly in inhibition of neoplastic cell proliferation. We found 28 genes involved in neoplastic cell proliferation are dysregulated in A431^SE1^ cells compared to A431^Ctrl^ cells. Among them, 16 genes were downregulated and 12 genes were upregulated in A431^SE1^ cells (Figure 3C). IPA software predicted dysregulation of oncogenic Akt pathways in A431^SE1^ cells compared to control cells. We found that 26 genes which are critically involved in Akt pathway were differentially expressed in A431^SE1^ cells compared to A431^Ctrl^. Out of these 26 genes, expression of 14 genes was reduced, with the highest reduction in the 6 genes, FOXO1, GAB1, EIF4EBP1, NFKBIA, and AKT1, and expression of 12 genes was increased, with the highest increased in the 6 genes ranked as CTNNB1, MAP2K2, RPS6KB1, JAK1, JAK2, and HSP90AB1 in A431^SE1^ cells compared to A431^Ctrl^ cells (Figure 3D). Taken together, the data from Kinex™ antibody array analyzed by IPA software conclude that CDC42SE1 overexpressing A431 cells inhibits cell proliferation by altering the Akt signaling pathway.

### 3.4. CDC42SE1 Inhibits A431 Cell Proliferation by Inhibiting Akt Pathway

Activated CDC42 (GTP bound CDC42) binds to the p85 subunit of PI3K leading to the activation of PI3K/Akt pathways in cancer [41]. In order to characterize the mechanism of CDC42SE1 mediated inhibition of A431 cell proliferation, we quantified total and phosphorylated Akt, PTEN, 4EBP1, and mTOR signaling protein levels in A431^Ctrl^, A431^SE1^, and A431^SE1-H38A^ by immunoblot.

**Figure 4:**
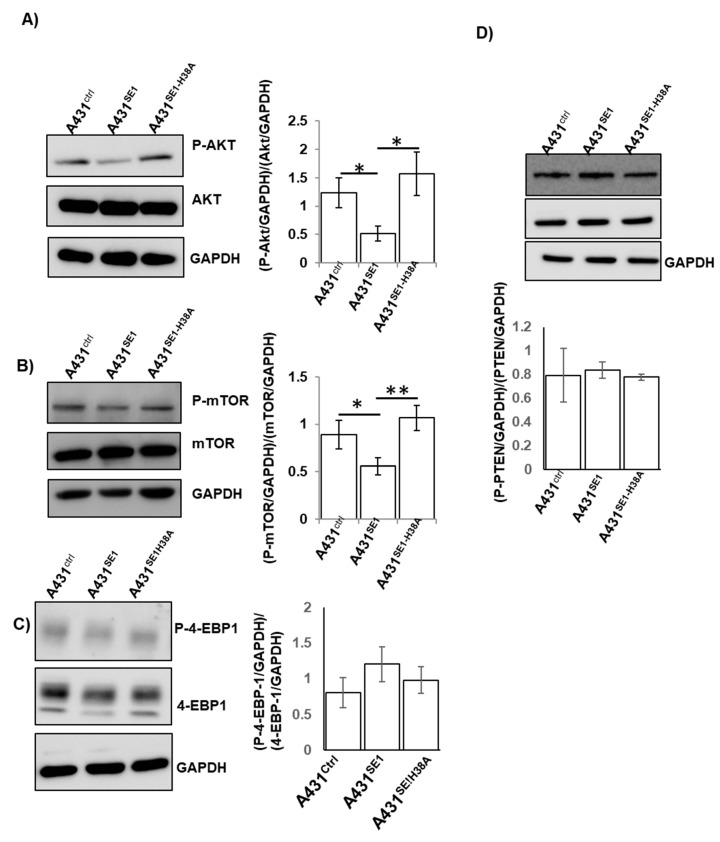
Akt pathway is inhibited in A431^SE1^ cells compared to A431^Ctrl^ cells. Protein lysate from A431^SE1^, A431^SE1-H38A^, and A431^Ctrl^ were subjected to western blot analysis using antibodies Akt, P-Akt (**A**), mTOR, P-mTOR, (**B**) 4-EBP1, (**C**) PTEN, P-PTEN (**D**), and GAPDH was used as a loading control (n = 3). The band intensities were quantified using ImageJ software and normalized using GAPDH and plotted. (** *p* ≤ 0.01, * *p* ≤ 0.05).

We found that relative levels of P-Akt and P-mTOR levels were lower in A431^SE1^ cells compared to A431^Ctrl^ and A431^SE1-H38A^, even though total Akt and mTOR levels were the same in all the three cell lines, suggesting that CDC42SE1 inhibits the activation of Akt and mTOR. Additionally, the overexpression of CDC42SE1 caused a reduction in the expression of 4-EBP1 in A431^SE1^ cells compared to A431^Ctrl^ cells (Figure 4A–E). The activity of Akt is downregulated by the phosphatase PTEN, which converts PIP3 to PIP2 [42]. No significant differences in the expression of PTEN was observed in A431^SE1^ and A431^Ctrl^ cells. These results suggest that reduction of Akt activity in A431^SE1^ cells was not due to increased PTEN expression. The reduced phosphorylation at position T308 in Akt (pT308) in A431 cells might be the result of the instability of p-Akt (pT308) or its dephosphorylation of pT308 by protein phosphatase 2A (PP2A) [43]. Thus, our results suggest that the overexpression of CDC42SE1 in A431 cells dysregulate the Akt signaling pathways, resulting in inhibited cell proliferation.

### 3.5. CDC42SE1 Localizes at the Plasma Membrane and Cytoplasm in A431 Cells

CDC42SE1 is a small scaffold protein and its overexpression caused membrane blebbing in NIH-3T3 cells but not in COS1 cells [14]. In order to characterize the localization of CDC42SE1 and its mutant CDC42SE1^H38A^ in A431 cells, we generated plasmid expressing GFP-tagged CDC42SE1 (pLJM-CDC42SE1-GFP) and CDC42SE1^H38A^ (pLJM-CDC42SE1^H38A^-GFP) using pLJM1-GFP [30].

**Figure 5:**
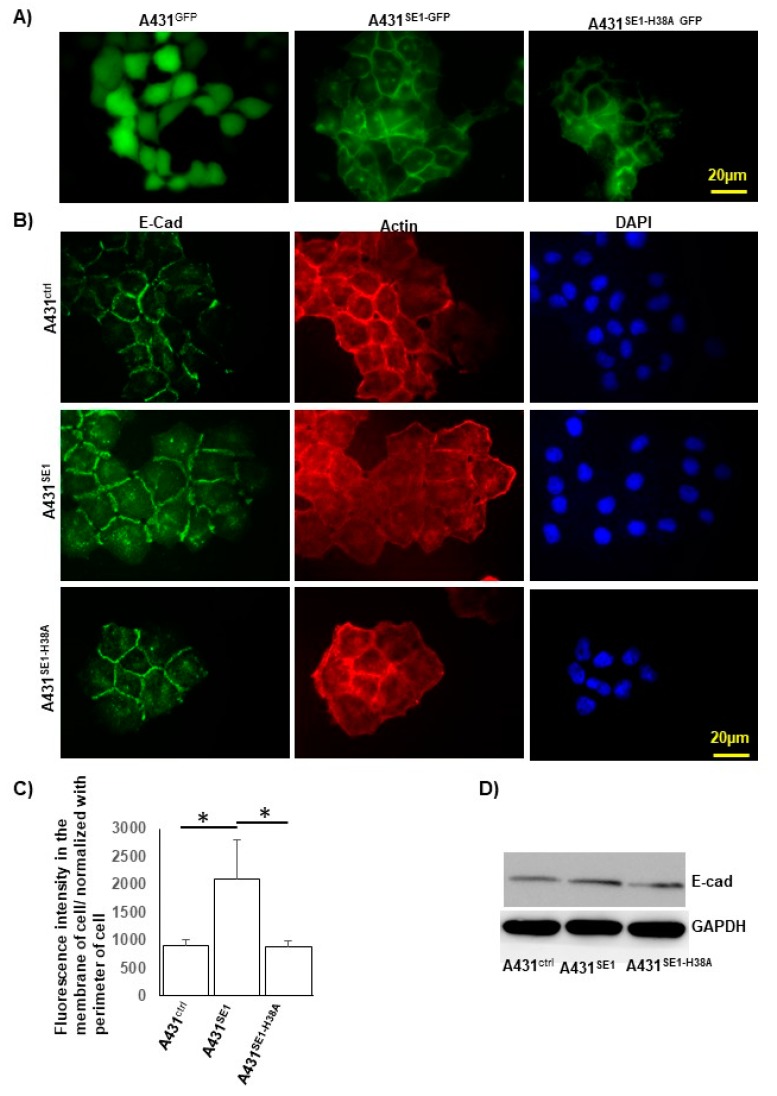
CDC42SE1 localizes at the plasma membrane and enhanced E-cadherin localization to the membrane in A431^SE1^ cells. (**A**) A431 cells were infected with lentivirus harboring expression cassette for CDC42SE1-GFP, CDC42SE1^H38A^–GFP, and GFP. The infected cells were visualized using a Olympus fluorescent microscope fitted with 40X oil objective. (**B**) A431^Ctrl^, A431^SE1^, and A431^SE1-H38A^ cells were seeded on coverslips, grown to 40% confluency, fixed, and probed with anti-E-cadherin primary antibody followed by Alexa488 secondary antibody. Alexa568-Phalloidin was used to visualize F-actin in cells. Images were taken using 40X objective. (**C**) Quantification of fluorescence intensity of E-cadherin localized in the membrane of A431^Ctrl^, A431^SE1^, and A431^SE1-H38A^ cells. The fluorescence intensity of the cell was normalized with the perimeter of the cells. (**D**) Protein lysate from A431^SE1^, A431^SE1-H38A^, and A431^Ctrl^ were subjected to western blot using antibodies E- cadherin. GAPDH was used as a loading control. (n = 3) (* *p* ≤ 0.05).

A431 cells were infected with the lentivirus expressing GFP, CDC42SE1-GFP, or CDC42SE1^H38A^-GFP to generate stable cell lines. The stable cell lines were seeded in 6-well plates with a coverslip, incubated for 24 h, the coverslip was subsequently placed on microscope slides, and the cells were imaged using a fluorescent microscope (Figure 5A). CDC42SE1-GFP and CDC42SE1^H38A^-GFP were found to be localized at the plasma membrane as well as in the cytoplasm of A431 cells. This suggests that CDC42SE1 can localize to the plasma membrane independently of its interacting partner CDC42, which localizes to the plasma membrane mediated by the lipid modification at the C-terminus [44]. To characterize the role of CDC42SE1 in cell-cell adhesion, we carried out immunostaining of A431^Ctrl^, A431^SE1^, and A431^SE1-H38A^ cells to visualize E-cadherin localization. We found that there is increased E-cadherin immunostaining in the cell boundary of A431^SE1^ cells compared to A431^SE1-H38A^ and A431^Ctrl^ cells (Figure 5B). Quantification of the fluorescence intensity of E-cadherin localized on the membrane of the cells using Image J software (n = 30) suggest significant increased localization of E-cadherin on the plasma membrane of A431^SE1^ cells compared to A431^SE1-H38A^ and A431^Ctrl^ cells (Figure 5C). The increased membrane localization was partly due to increased expression of E-cadherin in A431^SE1^ cells compared to A431^SE1-H38A^ and A431^Ctrl^ cells, as determined by the E-cadherin immunoblot (Figure 5D). We also measured the expression of ZO-1 and Claudin-5 (tight junction proteins) by immunoblot analysis, but no significant differences were observed for both proteins across the cell lines (Figure S2A). E-cadherin is a cell adhesion molecule involved in mediating cell-cell adhesion, which is further strengthened by the cytoplasmic tail of E-cadherin interacting with β-catenin to form β-catenin/cadherin complex [45]. Previous reports have suggested that decreased expressions of E-cadherin and accompanying easy cell dissociation are noted during the early stage of cancer development [46]. Upregulation of E-cadherin in various cancer cells inhibits invasion, metastasis, and deficiency of E-cadherin reduced the tumor cell differentiation [46]. These results suggest that upregulation of E-cadherin may be involved in inhibition of proliferation of A431^SE1^ cells. Thus, the results suggest that CDC42SE1 localized predominantly in the cell membrane may be involved in signal transduction, cell proliferation, membrane trafficking, cytoskeleton assembly, and cell motility [47].

### 3.6. Overexpression of CDC42SE1 Inhibits A431 Cell Spreading

Cell adhesion to ECM (Extra Cellular Matrix) is important to promote cell migration, cell adhesion, cell spreading, cell proliferation, and cytoskeleton organization [48]. Previously, we reported that reduced cell adhesion and increased cell motility correlate in A431 cells during both normaxia and hypoxia [49]. To identify the role of CDC42SE1 in A431 cell spreading, we seeded stable cells of A431^Ctrl^, A431^SE1^, and A431^SE1-H38A^ cells on fibronectin coated 96-well plate. Cells were imaged at 0, 10, and 20 min using DIC microscopy.

**Figure 6:**
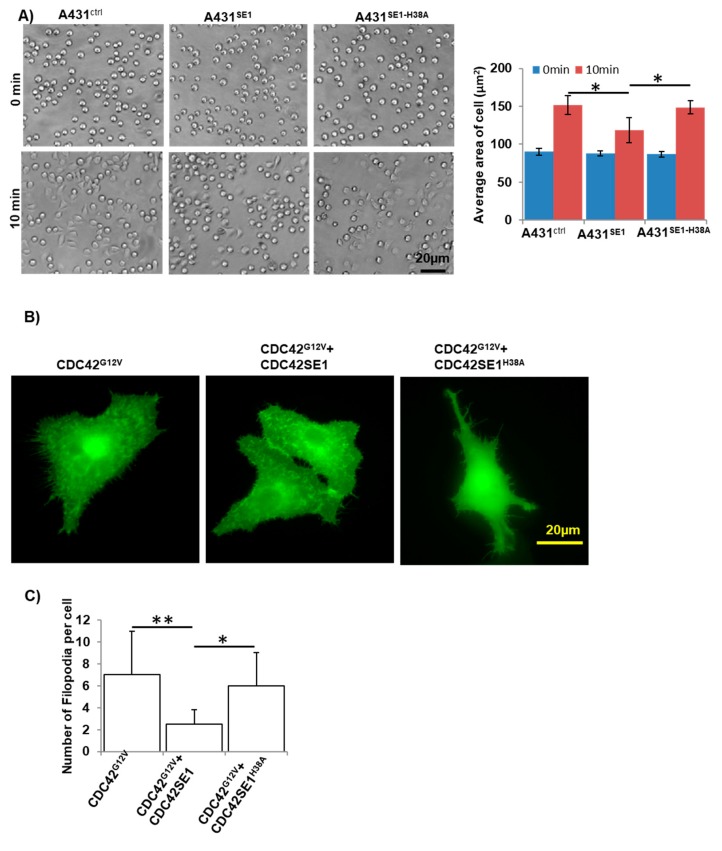
CDC42SE1 inhibits A431 cell spreading and CDC42 induced filopodia formation in A549 cells. (**A**) A431^Ctrl^, A431^SE1^, and A431^SE1-H38A^ cells were seeded on fibronectin coated 96 well plates and cells were imaged using microscope (10× objective) at 0, 10, and 20 min time interval. Images of 30 cells per sample were used to calculate the area of the cells using Image J (n = 3). (**B**) A549 cells were transfected with 4 µg of plasmid in the combinations, as shown in the figure. Cells were analyzed for the filopodia formation 36 h after transfections. The images were acquired using 40× objective lens. (**C**) A total of 30 transfected cells were selected at random fields and analyzed for the presence of filopodia using Image J software. We counted cells with filopodia when the cell protrusion was between 8–20 µm (n = 3). Results are mean ± SD ** *p* < 0.01, * *p* < 0.05.

A431^SE1^ cells spread slowly at 10 min compared to A431^Ctrl^ and A431^SE1-H38A^ cells (Figure 6A), but the cell spreading of all the cells were comparable at 20 min. This observation corroborates with our antibody microarray data analysis by IPA, which revealed that the cell spreading pathway of A431^SE1^ cells was attenuated compared to A431^Ctrl^ cells. This is due to alterations in various signaling protein, such as Akt, VIM, MET, FGFR1, and MARCKS that are important for cell spreading (Figure S2B). The yeast two hybrid and GST pulldown results revealed that CDC42SE1 interacts with active CDC42, which inhibits CDC42 induced JNK signaling in COS1 cells [8,14]. CDC42 together with its effector protein N-WASP are required for cell adhesion and spreading [48]. Thus, our cell spreading results and IPA analysis suggests that overexpression of CDC42SE1 inhibits the cell spreading by interfering with CDC42 regulated cell adhesion mediated by β1 integrin [48] in A431 cells. Competitive binding of CDC42SE1 to CDC42 possibly interferes with CDC42 effectors, resulting in the inhibition of CDC42-mediated A431 cells spreading.

### 3.7. Overexpression of CDC42SE1 Inhibits CDC42-Induced Filopodia Formation in A549 Cells

Filopodia are thin actin rich plasma membrane protrusions that extend out from the edge of the cell [11]. A previous study reported that filopodia-like protrusions are extensively used by cancer cells to migrate and invade into distant metastatic sites [50]. Formation of filopodium protrusions are driven by actin polymerization, which are regulated by the small GTPase, CDC42, actin capping proteins, and actin regulators [11]. CDC42 has been shown to play an important role in cancer cell migration and filopodia formation in various human cancers cells [7,11]. To test the hypothesis that binding of CDC42SE1 to CDC42 negatively regulates CDC42 activity in filopodia formation, we analyzed the filopodia induced by CDC42 in the presence and absence of CDC42SE1. The transfection efficiency was very low in A431 cells, thus we performed the filopodia analysis using A549 cells. We transfected plasmid expressing GFP and plasmid expressing dominant active CDC42 mutant, CDC42^G12V^, with either empty vector or plasmid expressing CDC42SE1 or CDC42SE1^H38A^. Cells were imaged 36 h post-transfection and the number of filopodia (8 and 20 μm) were counted manually. The number of filopodia per cell was significantly reduced in cells expressing CDC42^G12V^ and CDC42SE1 compared to cells only expressing CDC42^G12V^ (Figure 6B,C). These results suggest that CDC42SE1 inhibits CDC42 induced filopodia formation in A549 cells (Figure 6B,C). Cells expressing CDC42^G12V^ and CDC42SE1^H38A^ or CDC42^G12V^ alone had a comparable number of filopodia per cell (Figure 6B,C), suggesting that CDC42-CDC42SE1 interaction is necessary to attenuate filopodia formation.

### 3.8. CDC42SE1 Suppresses Growth of A431 Tumors In Vivo

**Figure 7:**
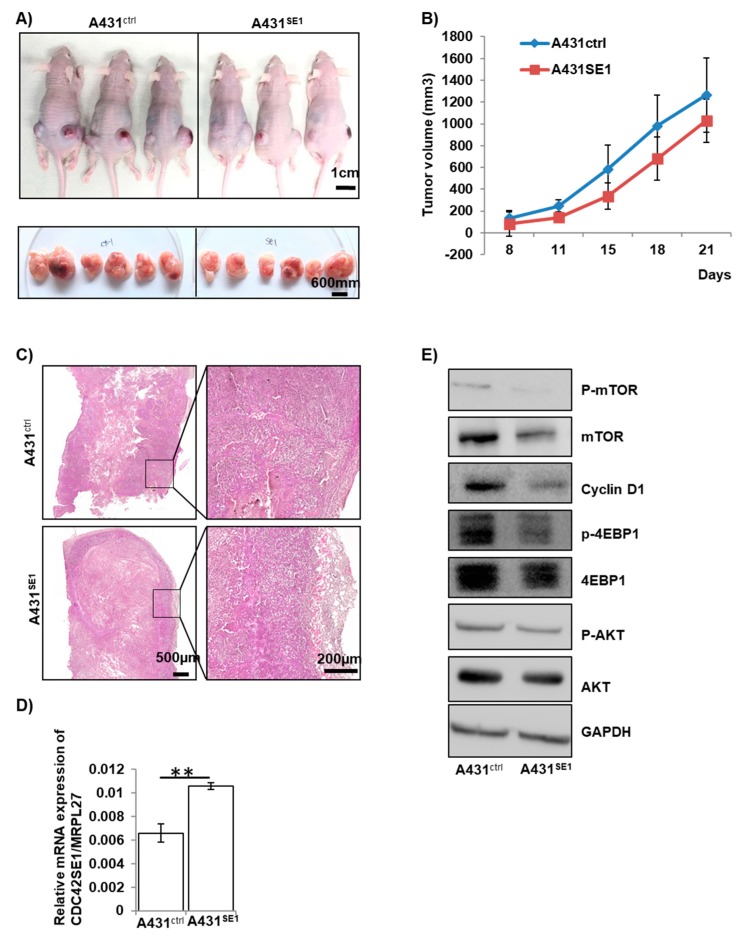
CDC42SE1 suppresses growth of A431-derived tumors in-vivo. (**A**,**B**) A431^Ctrl^ and A431^SE1^ cells (1 × 10^6^ cells) with 50% of matrigel were injected subcutaneously into nude mice (n = 6 for each group). The mice were photographed every two days and the image shown is of mice 21days after the injection. The tumor size was measured by Vernier caliper at 8th, 11th, 15th 18th, and 21st day after injection of A431cells into nude mice, and the tumor volume was calculated using L X W^2^/2 formula. Data points represent mean tumor volumes. (**C**) Tissue sections from mice injected with A431^SE1^ and A431^Ctrl^ cells were prepared and stained with H & E. H & E staining image showed that the tumors formed by A431^Ctrl^ cells were well organized and differentiated compared to tumors formed by A431^SE1^ cells (n = 6). (**D**) Total RNA was isolated from A431^Ctrl^ and A431^SE1^ tumor samples using Trizol, converted to cDNA, and used to perform qPCR with CDC42SE1 and MRPL27 specific primers. Relative abundance of CDC42SE1 was normalized against MRPL27 (n = 3). (**E**) Protein lysate from A431^SE1^ and A431^Ctrl^ tumors were subjected to immunoblot analysis using antibodies against Akt, P-Akt, mTOR, P-mTOR, 4-EBP1, and cyclin D1. GAPDH was used as a loading control (n = 3). (** *p* ≤ 0.01, * *p* ≤ 0.05).

The results from previous sections suggest that CDC42SE1 inhibits A431 cell proliferation in-vitro (Figure 1E,F). Thus, we asked if the overexpression of CDC42SE1 in A431 will affect the growth of A431 tumors in vivo using xenograft assay in nude mice. A431^SE1^ or A431^Ctrl^ cells mixed with matrigel (1 × 10^6^ cells in 50 μL of cold DMEM and 50 μL of matrigel) were injected subcutaneously into the nude mice (Figure 7A).

The tumor size derived from A431^Ctrl^ and A431^SE1^ was measured using a digital Vernier caliper on the 8th, 11th, 15th 18th, and 21st days post-injection into nude mice (n = 6) and plotted (Figure 7B). Both A431^Ctrl^ cells and A431^SE1^ cells formed tumors when injected into nude mice. However, the tumors formed by A431^SE1^ cells were smaller compared to the tumors formed by A431^Ctrl^ cells (Figure 7A,B) at all the time points that were recorded. We found that the volume of the tumor formed by A431^SE1^ cells was significantly reduced by 21% at the end of experiment, compared to the tumors formed from A431^Ctrl^ cells. Thus, the results suggest that CDC42SE1 overexpression in A341 cells inhibit xenograft tumor growth. In addition, H & E staining of the tumor sections showed that A431^Ctrl^ cells formed well-organized and differentiated tumors compared to the tumors formed by A431^SE1^ cells (Figure 7C). Immunoblot showed that the levels of P-Akt, P-4EBP-1, cyclin D1, and P-mTOR levels were decreased in A431^SE1^ tumors compared to A431^Ctrl^ tumors (Figure 7D,E), consistent with in vitro results (Figure 4A–E), suggesting that CDC42SE1 reduced tumorigenesis by inhibiting the Akt pathway. Angiogenesis, the process of forming new blood capillaries from pre-existing blood vessels, provides oxygen and nutrients for cell survival, proliferation of cancer cells, and tumor growth [51]. In order to characterize the role of CDC42SE1 in angiogenesis, we immunostained the tissue sections to visualize the endothelial cell marker CD31. A reduction of CD31 staining in A431^SE1^ cell tumor sections was observed compared to A431^Ctrl^ cell tumor sections (Figure S3). Previous studies reported that CD31 staining is an indicator of neovascular development and facilitates uncontrolled growth, invasion, and metastasis of cancer cells [51,52]. Taken together, in vitro and in vivo findings demonstrate that CDC42SE1 regulates the cancer cell proliferation and tumor formation. Thus, reduced expression of CDC42SE1 in skin cancer of patient samples may promote cell proliferation and tumorigenesis.

## 4. Discussion

Abnormal activation of Rho GTPases are associated with cancer proliferation, survival, migration, and metastasis [53]. CDC42 is a small GTPase involved in tumor initiation and cancer progression [13,54]. Previous reports have shown that CDC42 is overexpressed in various cancer tissues, including melanoma, colon cancer, and breast cancer [13]. Additionally, CDC42 knockdown induced cell cycle arrest and apoptosis in neuroblastoma cells [55]. These results suggests that CDC42 positively regulates the progression of various cancers [13]. CDC42 interacts with various downstream effector proteins via their CRIB or GBD domains to regulate cellular and biological functions. For instance, small scaffold protein CDC42SE1 interacts with CDC42 through its CRIB domain [8,14]. Previous reports have suggested that CDC42SE1 coordinate or mediate CDC42 induced cell shape changes in COS1 and NIH-3T3 cells [14]. The conserved CDC42SE1 CRIB domain consists of an α-helical C-terminal region, two conserved cysteine at the N-terminal, and basic amino acid preceding the CRIB domain that binds phosphoinositide [8]. Both the CRIB domain and α-helical region of CDC42SE1 is required for efficient binding to CDC42 as confirmed by pulldown experiment using GST-CDC42SE1 and it mutant with endogenous CDC42 produced in COS1 cells [14,15]. Phosphoinositide interacts with the basic amino acid of CDC42SE1, which may regulate CDC42SE1 activity at cell membrane and downstream signaling pathways [8,15]. These findings demonstrate that both the basic region and the CRIB domain of CDC42SE1 are essential for its ability to regulate cellular and biological activities.

CDC42SE1 overexpression in NIH-3T3 fibroblast led to membrane blebbing [14]. CDC42SE1 has been shown to inhibit CDC42-induced JNK activity and cell morphological alteration in COS1 cells [13,14]. CDC42SE1 plays a crucial role in CDC42 induced F-actin accumulation at the immunological synapse [8] and during phagocytosis [15]. The role of CDC42SE1 in cancer has not been characterized to date. First, we identified that the expression of CDC42SE1 was significantly reduced in human SCC samples (n = 5) compared to matched perilesional controls (n = 5). We also found that patients with reduced expression of CDC42SE1 had poor prognosis compared to patients with high expression of CDC42SE1 (Figure S1). Furthermore, we found that CDC42SE1 is highly expressed in normal keratinocytes (HaCaT), whereas its expression is reduced in carcinoma cell lines (A431 and HSC-5). These results suggest that expression of CDC42SE1 is reduced in skin cancer samples and skin cancer cell lines. Next, we carried out in vitro proliferation assays in A431^Ctrl^, A431^SE1^, and A431^SE1-H38A^ cells. MTT assay and proliferation assays revealed that A431^SE1^ cells have significantly reduced cell proliferation compared to A431^Ctrl^ cells, while A431^SE1-H38A^ cells showed cell proliferation comparable to A431^Ctrl^ cells, suggesting that the mutation which impaired CDC42SE1-CDC42 interaction impaired CDC42SE1 ability to attenuate cell proliferation.

In addition, overexpression of CDC42SE1 reduced colony numbers and size of A431^SE1^ cells compared to A431^SE1-H38A^ and A431^Ctrl^ cells in clonogenic assay, suggesting that CDC42SE1 inhibits tumor initiation and cell survival [56] in A431 cells compared to A431^Ctrl^ and A431^SE1-H38A^ cells. A431^SE1^ cells formed fewer and smaller colonies compared to A431^Ctrl^ and A431^SE1-H38A^ cells in soft agar colony formation assay, suggesting that the reduced size of the colony in A431^SE1^ cells could be due to the decrease in the growth rate caused by CDC42SE1 under anchorage-independent conditions [57]. We validated our in vitro results using in vivo tumorigenesis assays in nude mice and found that A431^SE1^ cells formed smaller tumors compared to A431^Ctrl^ cells, confirming the in vitro data—CDC42SE1 reduces cell proliferation. We propose that CDC42SE1 competes with other CDC42 effector proteins for binding to CDC42, resulting in CDC42s inability to perform various functions. CDC42SE1-CDC42 complex possibly recruits other proteins and inhibits cell proliferation.

To further characterize the role of CDC42SE1 in cell proliferation, we quantified cell signaling proteins using antibody microarray analysis. The data suggest that the Akt pathway is attenuated by the overexpression of CDC42SE1, as 26 genes which are critically involved in Akt pathway were differentially expressed in A431^SE1^ cells compared to A431^Ctrl^ (Figure 3). The antibody microarray and IPA analysis was verified by immunoblot analysis, which revealed reduced levels of P-Akt, P-mTOR, and cyclin D1 in A431^SE1^ cells compared to A431^Ctrl^ and A431^SE1-H38A^ cells. These results suggest that CDC42SE1 inhibits proliferation of A431 cells by inhibiting of Akt pathway. A previous report mentioned that CDC42 has a cancer promoting role under the control of Ras signaling pathway and also CDC42 binds to p85 regulatory subunit of PI3K to active the downstream Akt signaling pathway, which are highly activated during cancer cell proliferation [41]. CDC42 knockdown revealed a significant reduction in phosphorylation of Akt found in HRas^G12V^ transformed cells, however there was no alteration in MEK and P-ERK pathways [58]. Phosphorylation at Thr308 or Ser473 of Akt has a crucial role in the activation of PI3K/Akt pathways in SCC and BCC skin samples [22]. In the cancer patient samples, it was shown that activated Akt promotes protein synthesis in cancer cells through mTOR complex [27]. These results suggest that the Akt pathway plays a crucial role in skin cancer, which can be modulated by activated CDC42 signaling pathway. PTEN is a tumor suppressor and is highly expressed in several normal human tissues, such as liver, brain, and placenta [42]. Decreased expression of PTEN or point mutation in PTEN have been observed in various cancers, such as glioma, lung cancer, liver cancer, and endometrial cancer [59]. A previous study has shown that Akt is a downstream target of PDK1, which phosphorylates and activates Akt in various cancers. Constitutive activation of PI3K/PDK-1/Akt signalling pathway could promote cancer cell survival, tumor growth, and resistance to the anticancer drug in many human cancer cells [27]. We observed no significant changes in the expression of PTEN in A431^SE1^ cells compared to A431^Ctrl^ cells. These results suggest that reduction of Akt activity in A431^SE1^ cells was not due to changes in PTEN expression. Reduced P-Akt (pT308) in A431 cells could be due to the reduced stability of p-Akt (pT308) or the dephosphorylation of pT308 by protein phosphatase 2A (PP2A) [43]. Thus, we propose that overexpression of CDC42SE1 reduced the available CDC42 pool in the cell resulting in the inhibition of Akt. The antibody microarray also revealed downregulation of other oncogenic signaling molecules, such as PAK, EGFR, and Src in A431^SE1^ cells to control cells. This result indicates that downregulation of this oncogenic protein signaling along with Akt pathway might be involved in the reduction of proliferation of A431^SE1^ cells.

Several studies indicate that cell shape and cell spreading are necessary for efficient cell cycle progression [60]. Cell adhesion to the extracellular matrix (ECM) stimulates integrin-mediated signaling, thus inducing cell proliferation [61]. In NRK cells, inefficient cell spreading leads to reduced proliferation [60]. Interaction of integrins with the extracellular matrix (ECM) proteins promotes the remodeling of the actin cytoskeleton by regulating Rho GTPases, such as CDC42, which transduce signals from ECM to perform various cellular processes, including cell spreading and proliferation [48]. In this study, we found that CDC42SE1 inhibits cell spreading on a fibronectin coated surface. Previous reports mentioned that CDC42 and its effector proteins play an important role in cell adhesion and cell spreading [48]. Thus, overexpression of CDC42SE1 probably reduced the available CDC42 pool in the cell, resulting in poor cell spreading.

In summary, we found that the expression of CDC42SE1 was reduced in human skin cancer samples compared to controls; similarly, we also found that the expression of CDC42SE1 was reduced in skin cancer cell lines, and A431 cells compared to normal keratinocytes HaCaT cells. Overexpression of CDC42SE1 in A431 cells caused significant reduction in cell proliferation, colony size in soft agar, and tumor size in xenograft nude mice compared to A431^Ctrl^ cells. The reduced cell proliferation correlates with attenuation of AKT pathways, caused by CDC42SE1 interaction with CDC42 via the CRIB domain. Thus, our study has shown that downregulation of CDC42SE1 expression in cancer promotes tumorigenesis, and may be a novel marker for skin cancer development and progression.

## Supplementary Materials

The following are available online at http://www.mdpi.com/2073-4409/8/2/117/s1, Figure S1: CDC42SE1 expression in Head-neck squamous cell carcinoma using Kaplan-Meier Plotter, Figure S2: Overexpression of CDC42SE1 in A431 cells does not affect expression of tight junction proteins, Figure S3: CDC42SE1 expression decreased angiogenesis in xenograft tumor.
