# Supplementary material for: Overexpression of CDC42SE1 in A431 Cells Reduced Cell Proliferation by Inhibiting the Akt Pathway
Source: Cells. 2019 Feb 2;8(2):117. doi: 10.3390/cells8020117 (PMC6406378; doi:10.3390/cells8020117)
Supplement: Supplementary file 1 [file cells-08-00117-s001.pdf]

Supplementary Figures

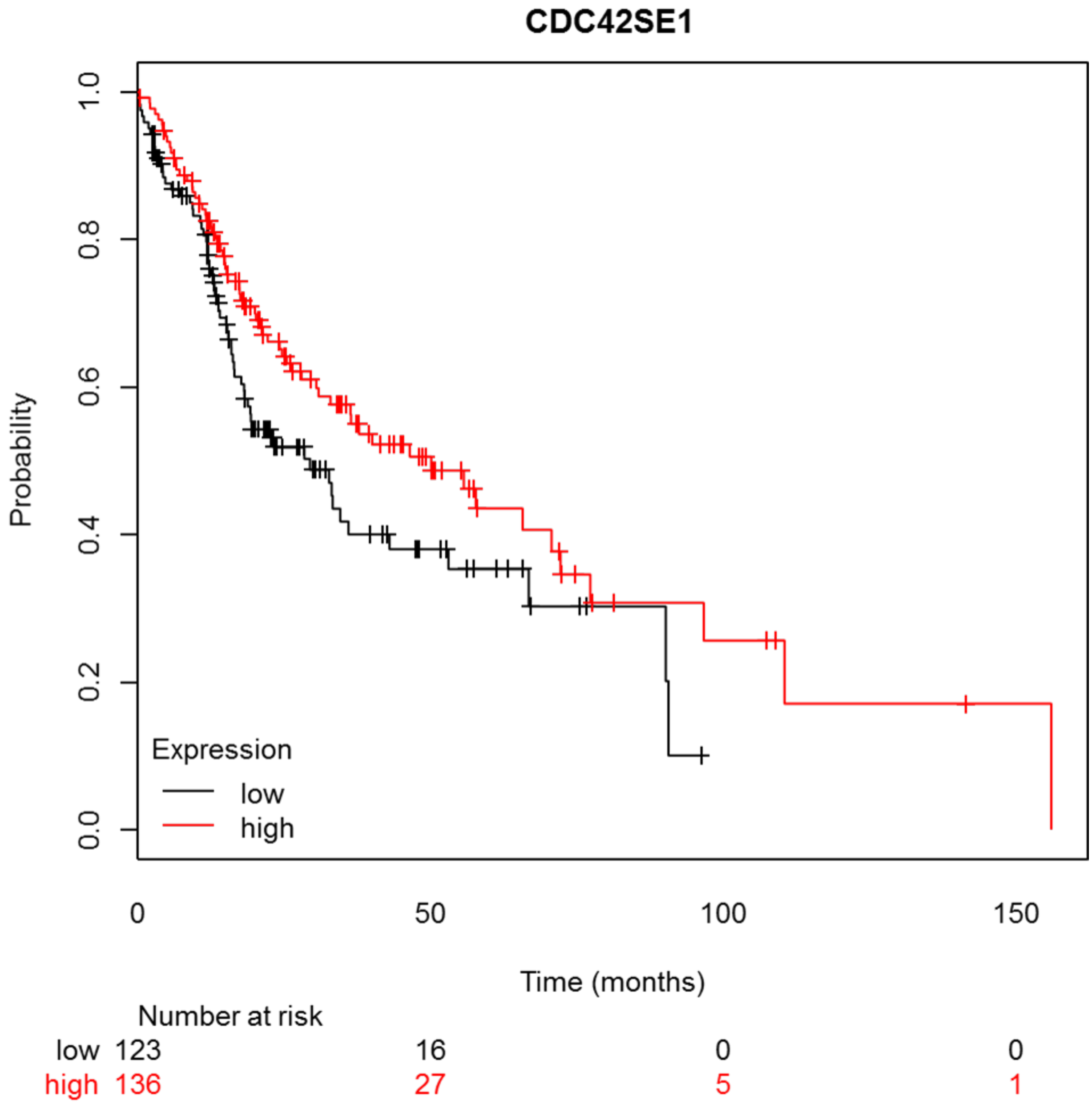

Figure S1: CDC42SE1 expression in Head-neck squamous cell carcinoma using Kaplan -Meier Plotter. The Plot showing overall survival in Head-neck squamous cell carcinoma. In Red; patient with expressions above the median, Black; patient with expressions below the median. CDC42SE1, p=0.0043

A)

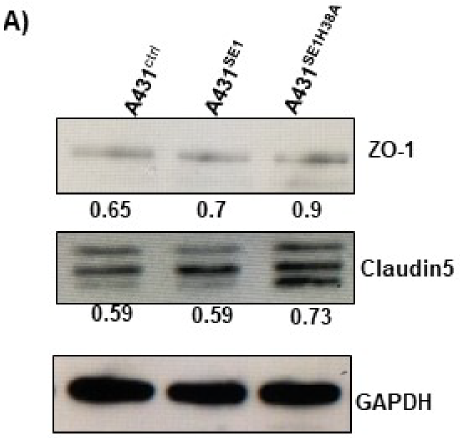

B)

SRC	-13.1127
PLCG1	-2.56565
MAPK14	-2.43698
PLCG2	-2.17845
MET	-1.93527
CDC42	-1.89021
PXN	-1.83819
AKT1	-1.82349
PAK1	-1.81416
RB1	-1.74907
STAT3	-1.68795
IRAK1	-1.58868
MARCKS	-1.46742
EGFR	-1.38462
MAPK3	-1.31449
SYK	-1.29149
IKBKG	1.20199
PAK3	1.242262
TP53	1.437394
CBL	1.541706
FES	1.640862
PTK2	1.764777
PRKCQ	1.809953
JAK2	1.826377
RPS6KB1	2.053662
PRKD1	2.05636
PTPN11	2.319252
MAP2K2	2.322556

Figure S2: Overexpression of CDC42SE1 in A431 cells does not affect expression of tight junction proteins

(**A**) Protein lysate from $A431^{Ctrl}$, $A431^{SE1}$ and $A431^{SE1\text{-}H38A}$ were subjected to western blot using antibodies against ZO-1 and Claudin-5. GAPDH was used as a loading control (n=3). (***$p \leq 0.001$, **$p \leq 0.01$, *$p \leq 0.05$). (**B**) The heat map represents genes implicated in spreading as was predicted to be inhibited by IPA software using Kinex™ data of protein lysate of $A431^{SE1}$ and $A431^{Ctrl}$.

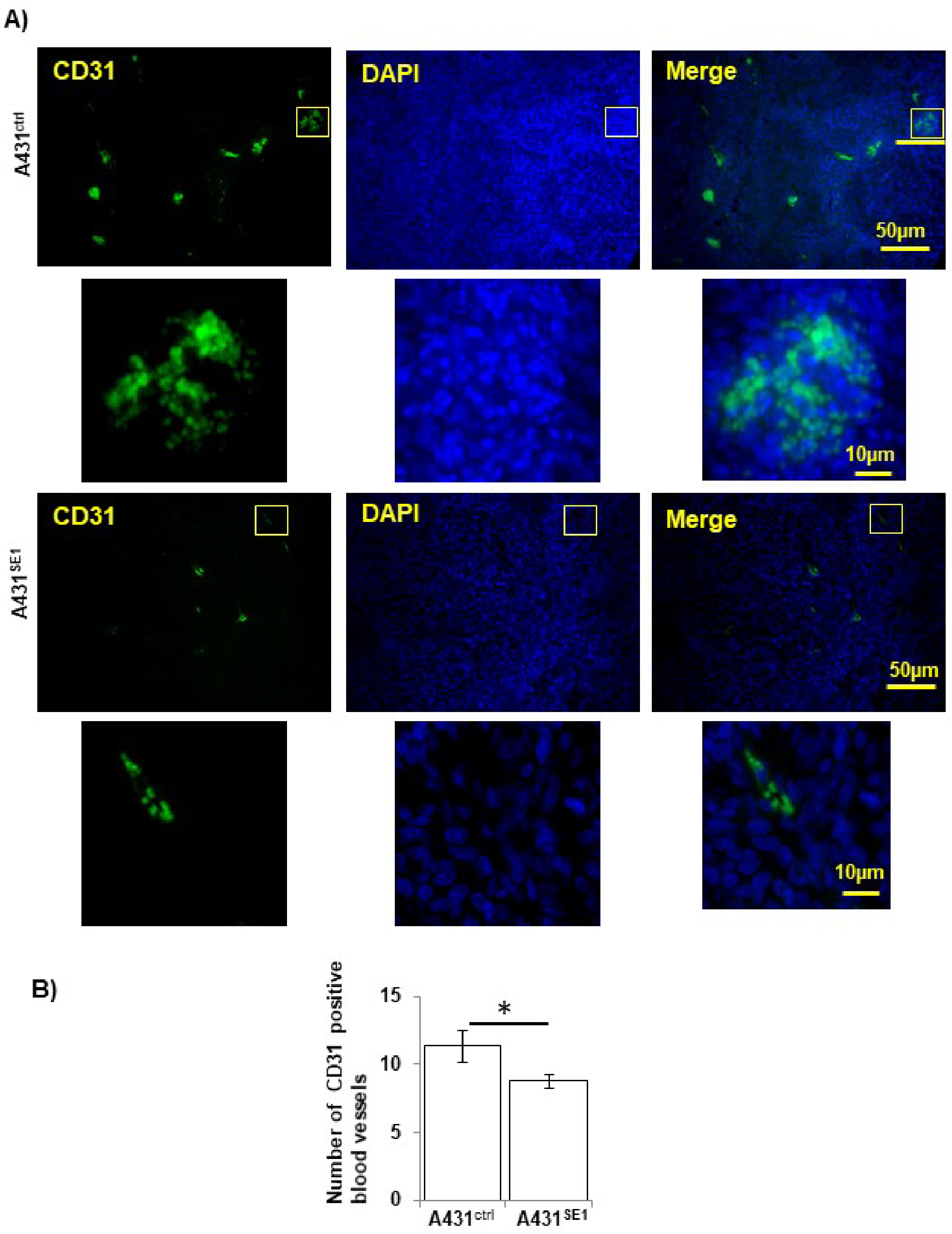

Figure S3: CDC42SE1 expression decreased angiogenesis in xenograft tumor

Tumor tissue sections from the A431^{Ctrl} and A431^{SE1} cells injected mice were prepared and probed with anti-CD31 (blood vessel endothelial cells marker) and subsequently with Alexa488 secondary antibodies to visualize expression of CD31 in tumor sections. The images were taken with 40X objective lens. Arrows indicate CD31 positive cells (n=6). The positive staining of CD31 in A431^{Ctrl} and A431^{SE1} tumor sections were manually counted at 3 random fields as shown in figure. Results are mean ± SD *** $p<0.001$, ** $p<0.01$, * $p<0.05$.
